# Therapeutic transjejunal endoscopy for the treatment of biliary complications after choledochojejunostomy

**DOI:** 10.3892/etm.2012.815

**Published:** 2012-11-19

**Authors:** GUO-PING LIU, WEN-XI ZHU, GUANG-MING CHENG, SHU-REN MA

**Affiliations:** 1 Department of General Surgery, Changhai Hospital, The Second Military Medical University, Shanghai 200433;; 2 Faculty of Nursing, Advanced Professional Technology College of China Medical University, Shenyang 110011;; 3 Departments of Hepatobiliary Surgery, Shenyang North Hospital, Shenyang 110015, P.R. China; 4 Endoscopy, Shenyang North Hospital, Shenyang 110015, P.R. China

**Keywords:** jejunum, endoscope, biliary intestinal anastomosis, jejunostomy

## Abstract

The present study aimed to assess the value of endoscopic jejunostomy for post-biliary intestinal anastomosis biliary complications. The clinical data of the endoscopic therapies by jejunal approach for post-biliary intestinal anastomosis biliary complications in 13 patients (16 surgeries in total) were retrospectively analyzed. The surgical success rate was 100% (16/16). Nasobiliary tube detention was performed for 2 patients, plastic stent placement for 5 and biliary metal stent placement for 4. The remaining two patients did not retain any drainage tube or bracket after surgery. The incidence rate of intraoperative anastomotic stenostomia was 76.9% (10/13). A noticeable postoperative decrease in bilirubin levels was observed in 10 patients. The level of gallstone-free patients was 75% (3/4). There were 10 cases in which cholangitis remission or no attack was identified. Post-operative incisional infection occurred in 3 patients, hepatophyma in 1 and an intestinal fistula in 1. Endoscopic therapy by jejunal approach for post-biliary intestinal anastomosis biliary complications has the virtue of being safe, effective and minimally invasive. It has extensive potential applications in clinical practice.

## Introduction

At present, cholangiojejunostomy mostly uses Roux-en-Y anastomosis. Broadly speaking, cholangioenterostomy in Whipple surgery is also within this category (1). Following cholangioenterostomy, complications, including biliary tract infection (2,3), lithogenesis (4) and obstructive jaundice (5) usually occur. The generation of these complications is mostly associated with anastomotic benign strictures (6) or tumor recurrence congestion, duodenal-biliary reflux or biliary-jejunal loop dyskinesis (7), as well as the increase in non-conjugated cholic acid and bacterial content in the jejunum following bilioenteric Roux-en-Y anastomosis (8,9). However, bilioenteric anastomotic strictures are the main pathological and anatomical causes generating these complications. In view of this, certain scholars have attempted to modify the bilioenteric Roux-en-Y anastomosis method by, for example, taking anti-reflux measures, but the result was unsatisfactory (10). A conservative treatment is also mostly ineffective. In conventional surgery, the trauma of treatment is greater and operating is more difficult. Also, a second, or even multiple, surgeries are required, which not only increases the surgical risk and difficulty, but also makes the bilioenteric anastomotic stoma narrower and causes further complications. Therefore, the efficiency of surgical treatment is unpredictable. Moreover, conventional peroral endoscopic therapy mostly fails due to changes in anatomical relationships caused by the post-operative gastrojejunal reconstruction. Although there are reports of successful procedures (11), the demands on the surgeons and equipment are higher and it is therefore difficult to encourage further promotion and application of this procedure. Previous studies have shown percutaneous and transhepatic balloon dilation of bilioenteric anastomotic strictures (12–14). Although an improved treatment efficiency is obtained, it is mostly necessary to indwell the drainage tube *in vivo* synchronously, which reduces the patient’s quality of life. Additionally, if there is calculus of the bile duct near to the anastomotic stoma, it is likely to take longer to complete the endoscopic treatment via this pathway. Consequently, it is of vital importance to seek a more effective and minimally invasive treatment method. To solve this problem, transjejunal endoscopy for the treatment of biliary complications following choledochojejunostomy is adopted. Based on summaries of the clinical experiences and observations of the treatment efficacy, a better evaluation of the safety and efficiency of this treatment method is required.

## Patients and methods

### General data

In total, 13 patients, including 6 males and 7 females, with a mean age of 59 years (range, 32–81 years) were included in this study. The study was conducted in accordance with the declaration of Helsinki and with approval from the Ethics Committee of Shenyang North Hospital. Written informed consent was obtained from all participants. Bilioenteric Roux-en-Y anastomosis was conducted in4 cases due to bile duct cancer in the porta hepatis, in 1 case due to congenital biliary duct cyst canceration, in 2 cases due to congenital biliary duct cysts and in 1 further case due to iatrogenic biliary duct injury. Palliative resection of bile duct cancer combined with biliary-jejunal loop anastomosis was conducted in 1 case due to cancer in the middle segment of the bile duct. Pancreaticoduodenectomy (PD) was conducted in 3 cases due to cancer in the lower segment of the common bile duct and in 1 other case due to ampulla cancer. Of these 13 cases, there were 8 cases of patients whose bilioenteric drainage tubes were indwelled at the bilioenteric anastomotic stoma and were led out from the lateral abdominal wall via a biliary-jejunal loop, 1 case of a patient whose inner pipe was led out from the bile duct at the porta hepatis and 4 cases of patients without drainage tubes. In addition, 1 patient required surgery twice, while another required 3 surgeries in total to complete the treatment. Following cholangioenterostomy, biliary complications occurred between 12 days and 20 years post-surgery, with a median time of 12 months. Moreover, 5 cases presented with upper-right abdominal distension and discomfort, 11 cases presented with recurrent chills and high fever and 11 cases presented with icterus.

### Imaging examination

Color Doppler ultrasound examination revealed that 10 cases presented with intrahepatic cholangiectasis and that 4 cases presented with calculus of the bile duct at the porta hepatis. Of these, 3 cases presented with intrahepatic secondary bile ducts complicated by calculus.

### Surgical approach and endoscopic technique

**Figure 1.**
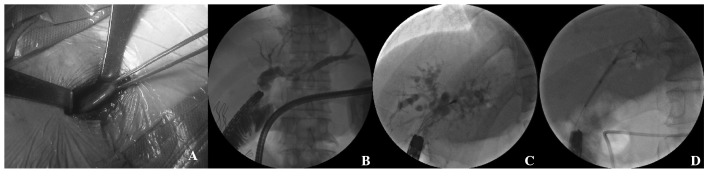
Surgical approach and endoscopic technique. (A) A nasobiliary duct installation in the biliary-jejunal loop as a marker. (B) Cholangiography. (C) Basket extraction of a stone. (D) Metal biliary stent installation in the biliary tract.

The surgical approach was to seek the biliary-jejunal loop. Following systemic anesthesia, the skin scar was led out from the original bilioenteric inner pipe using the lateral abdominal wall as the midpoint, and the skin was longitudinally cut open (3 to 5 cm) and deepened layer by layer to find the biliary-jejunal loop in the abdomen. The biliary-jejunal loop was mostly attached to the lateral peritoneum, its marker being the residual line junction fixed onto the lateral peritoneum by suturing. Subsequently, the contralateral mesentery wall of the biliary-jejunal loop was cut open, and purse-string sutures were performed on the seromuscular layer around the incision in order to provide a channel for the endoscopic technique. The bilioenteric inner pipe of 1 case was not led out from the biliary-jejunal loop. In the cases of the 4 patients without drainage tubes, the incision was made in the upper right abdomen via the rectus abdominis, and a nasobiliary duct was installed in the biliary-jejunal loop as the marker for seeking this loop (Fig. 1A).

The endoscopic technique was an exploration first conducted with an ultrafine gastroscope to find the bilioenteric anastomotic stoma, while under the coordination of the digital subtraction technique (DST). Subsequently, a gastroscopy was conducted to carry out the procedures required, including cholangiography, basket extraction, balloon dilatation or biliary stent installation (Fig. 1B–D). Of these cases, the bilioenteric anastomotic stoma of 1 patient was completely occluded by a cancer embolus due to bile duct cancer metastasis, and so it was impossible to insert a guide wire via the anastomotic stoma under the endoscope. Therefore, percutaneous transhepatic biliary drainage (PTCD) was performed instead, and a Zebra guidewire was implanted via a PTCD tube and inserted into the biliary-jejunal loop via the anastomotic stoma. Next, an inner pipe was installed via the guide wire by means of the gastroscope. Finally, the internal and external dual drainage of the biliary tract was completed.

The biliary-jejunal loop was sutured in a total of 11 cases and was fixed onto the lateral abdominal wall. A drainage tube was implanted into the biliary-jejunal loop in 2 cases and then led out from the incision prior to being sutured. At the early post-operative stage, the dressing on the incision required changing daily and anti-inflammation and liver-protection therapy was conducted.

## Results

### General data

During the surgeries, metal stents were installed in 4 cases, plastic stents were installed in 5 and nasobiliary ducts were installed in 2.

### Surgical efficiency

In total, there were 9 patients that did not suffer post-operative cholangitis attacks, 2 patients whose post-operative cholangitis attacked at an early stage and eased at a later stage and 2 patients whose post-operative cholangitis was recurrent and persistent. With regard to the post-operative bilirubin levels, 10 cases showed a significant decrease, 2 cases demonstrated fluctuations and an slight increase and 1 case demonstrated fluctuations and a significant increase. With regard to the cholelithiasis treatment, 3 cases were successfully treated, with an overall stone clearance rate of 75% (3/4). Post-operative complications included 1 case of a jejunal fistula, 1 case with hepatapostema and 3 cases with incision infections. The incidence rate of intra-operative biliary-jejunal anastomotic strictures was 76.9% (10/13). A total of 16 surgeries were performed on 13 patients, with an overall success rate of 100% (16/16).

## Discussion

The majority of complications following cholangioenterostomy occur in the post-operative long term (15), with only a minority occurring in the post-operative short term (16). In the present study, 2 cases occurred within 2 months of surgery, 1 case after 3 months and the remaining cases all occurred after >6 months and were possibly associated with strictures caused by gradual scar tissue formation at the bilioenteric anastomotic stoma. Among the cases in this group, the incidence rate of intraoperative biliary-jejunal anastomotic stricture was 76.9% (10/13).

The results of the present study identified that the endoscopy treatment of biliary complications after choledochojejunostomy via the biliary-jejunal loop stoma has a high success rate and satisfactory short-term treatment efficiency, with no severe post-operative complications. This not only suggests that this method is safe and effective, but that it may also be used repeatedly in the same patient with minimal invasion. The successful implementation of this method further supports the currently advocated treatment model for biliary complications following cholangioenterostomy, namely endoscopy on the behalf of minimally invasive treatment methods (17). Although a previous study has also reported endoscopic stone extraction techniques via the jejunal loop (18), the primary diseases involved in these cases were calculi of the bile duct, while the primary diseases in the present study are malignant tumors. Generally, the jejunal loop in the reported cases was placed in an uncertain location in the subcutaneous tissue and the jejunal approaches were mostly preset, while the biliary-jejunal loop in the present study was mostly located in the abdominal cavity. Additionally, a fiber choledochoscope was mostly applied in the previous studies, while only gastroscopy and ultrafine gastroscopy were used in the cases from the present study. Also, biliary stents and nasobiliary ducts were installed in the majority of cases in the present study. At this time, there is no comparable research literature to this group of cases.

The biggest difficulty of transjejunal endoscopy for the treatment of biliary complications after choledochojejunostomy is how to accurately select the biliary-jejunal loop. As current complications mostly occur in the post-operative long term, the bilioenteric inner pipe is removed and the original abdominal wall sinus ostium is closed. In addition, the original drainage tube of the abdominal cavity is mostly led out from the right lateral abdominal wall. The two abdominal wall outlet scar locations are extremely close, which often causes difficulties in selecting the correct abdominal wall incision. Therefore, it is necessary not only to review the original surgical records, but also to request a detailed medical history in order to determine the outlet position of the original bilioenteric inner pipe at the abdominal wall, and thus correctly select an incision site. In the present study, 2 cases presented with complications in the post-operative short term and retained the bilioenteric inner pipe. It was therefore relatively easy to select the biliary-jejunal loop. However, the bilioenteric inner pipe of 1 case was led out via the bile duct at the porta hepatis, while 4 cases had no indwelling drainage pipes at all. Therefore, the biliary-jejunal loop was not fixed onto the abdominal wall, and it was extremely difficult to find. To avoid searching blindly, we installed a nasobiliary duct in the biliary-jejunal loop as a marker for finding it. After making an incision into the peritoneum, the jejunal tube which adheres to the lateral abdominal wall is visible, but an incision cannot be made without careful consideration. Generally, the biliary-jejunal loop is identified according to the residual line junction fixed onto the lateral peritoneum by biliary-jejunal loop suturing and also judged according to the relationship of the jejunal loop with the first porta hepatic. Afterwards, an incision is made into the contra-lateral mesentery wall of the biliary-jejunal loop to conduct a endoscopic examination for the final confirmation. Usually, an ultrafine gastroscope is used to first explore the position of the bilioenteric anastomotic stoma and a guide wire is indwelled. Subsequently, a common gastroscope is used to conduct the surgery. As the jejunal tube is tortuous and adherent, the gastroscope entry direction is usually inconsistent with the upper and lower direction of the biliary-jejunal loop under the incision. It is feasible to successfully find the bilioenteric anastomotic stoma only under the guidance of DST. Certain studies (19–21) showed that it was feasible to determine the biliary-jejunal loop position under the guidance of ultrasound, CT or X-ray fluoroscopy, but this relies on the jejunal loop being located in the subcutaneous tissue or under the anadesma, and its application has certain limitations.

Endoscope entry via the percutaneous jejunostomy pathway usually causes an air leakage phenomenon, particularly at the early stage of endoscope entry, which causes interference in the endoscopic field of vision. Therefore, purse-string suture is performed on the seromuscular layer around the endoscope. As the endoscope enters, appropriate tightening is conducted and the jejunal wall stoma is surrounded with gauze so that a better result may be obtained.

In the present study, we initially injected air into the enteric cavity to increase the field of vision in 2 cases. Severe pneumatosis occurred in the enteric cavity during surgery and post-operative abdominal distension of the patients was apparent. This was not only unfavorable for abdominal closure, but also increased the risk of intestinal fistulas or incision infections. Even if gastrointestinal decompression is conducted, it is difficult to relieve these symptoms. To avoid this phenomenon, we replaced the injected air with CO_2_. In addition, the gas in the enteric cavity, particularly at the distal end of biliary-jejunal loop, was drawn out as much as possible following the completion of surgery.

To locate the bilioenteric anastomotic stoma, patience is required. As the bilioenteric anastomotic stoma may suffer from an inflammatory stricure or recurring tumor congestion, finding it becomes difficult even if the endoscope entry direction is correct. It may be necessary to repeatedly change between gastroscope, duodenoscope and ultrafine gastroscope. Also, multiple attempts using radiography are required to try to insert the guide wire in order to find the biliary tract. In the present study, the bilioenteric anastomotic stoma was occluded in 1 patient due to tumor recurrence, and it was impossible to insert the guide wire from the enteric cavity into the bile duct. Therefore, PTCD was conducted under the guidance of Color Doppler ultrasound. The guide wire was inserted via the PTCD tube and led into the biliary-jejunal loop through the bile duct at the porta hepatis and the bilioenteric anastomotic stoma. Inside the enteric cavity, the guide wire was captured with biopsy forceps. The biliary stent was passed along the guide wire and implanted to complete the internal and external dual drainage system. Following surgery, an efficient reduction in Bilirubin levels provided good evidence of the coexistence of internal and external drainage. Also, washing the PTCD tube removed obstructions from the the biliary stent. Therefore, due to simple PTCD external drainage, a majority of the bile was now able to enter the intestinal tract and injury to digestive function was avoided. The patient now maintains a good appetite.

With the exception of 2 patients who suffered from complications in the early post-operative stage who retained the bilioenteric inner pipe, and for whom biliary-jejunal loop ostomy was conducted following therapeutic endoscopy, the primary suturing of the intestinal loop incision was conducted for the remaining cases. The suture position was fixed onto the peritoneum under the incision. Of these cases, only one patient sufferred from an intestinal fistula, which was associated with poor nutritional status or malnutrition and was not medicated in time due to economic reasons.

To avoid post-operative incision infections, it is necessary to use an aspirator to absorb the intraoperative outflow of intestinal juice. Also, the incision must be thoroughly disinfected prior to abdominal closure. Following surgery, dressings must be changed regularly and subcutaneous effusion monitored. If necessary, active drainage and simultaneous physiotherapy may be carried out. The prevention of incision infections also contributes to the prevention of intestinal fistulas. In the present study, 3 cases presented with incision infections of varying degrees. Following drainage and physiotherapy, the symptoms disappeared. As a result of these accumulated experiences, later cases did not suffer from incision infections.

Transjejunal endoscopy for the treatment of biliary complications after choledochojejunostomy is a further development of endoscopic minimally invasive technology. It is an effective minimally invasive method for the control of icterus and cholangitis symptoms in particular, and for certain patients with advanced tumors or for those in which repeated surgeries are unsuitable. Although it is necessary to accumulate further cases and constantly generate summaries and improvements, this method is worthy of further promotion and application due to its improved treatment efficiency and technological advantages.
